# Sulfocoumarins as dual inhibitors of human carbonic anhydrase isoforms IX/XII and of human thioredoxin reductase

**DOI:** 10.1080/14756366.2020.1712596

**Published:** 2020-01-13

**Authors:** Mikhail Krasavin, Raivis Žalubovskis, Aiga Grandāne, Ilona Domračeva, Petr Zhmurov, Claudiu T. Supuran

**Affiliations:** a Department of Chemistry, Saint Petersburg State University, Saint Petersburg, Russian Federation; b Latvian Institute of Organic Synthesis, Riga, Latvia; c Faculty of Materials Science and Applied Chemistry, Institute of Technology of Organic Chemistry, Riga Technical University, Riga, Latvia; d Neurofarba Department, Universita degli Studi di Firenze, Florence, Italy

**Keywords:** Anticancer agents, carbonic anhydrase inhibition, thioredoxin reductase inhibition, hypoxia, oxidative stress

## Abstract

The hypothesis that sulfocoumarin acting as inhibitors of human carbonic anhydrase (CA, EC 4.2.1.1) cancer-associated isoforms *h*CA IX and – *h*CA XII is being able to also inhibit thioredoxin reductase was verified and confirmed. The dual targeting of two cancer cell defence mechanisms, i.e. hypoxia and oxidative stress, may both contribute to the observed antiproliferative profile of these compounds against many cancer cell lines. This unprecedented dual anticancer mechanism may lead to a new approach for designing innovative therapeutic agents.

## Graphical Abstract

**Figure:**
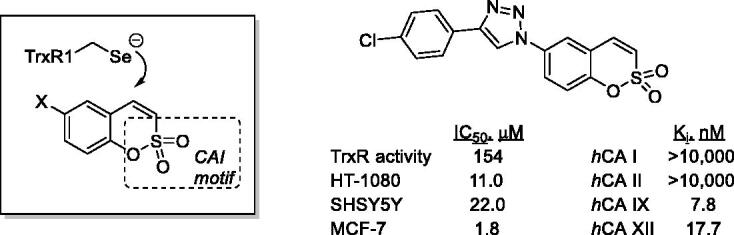

## 1. Introduction

Earlier, we reported 6-substituted sulfocoumarins **1**^1^ (designed as isosteres of the structurally related coumarins^2–6^) as potent and remarkably isoform-selective inhibitors of the metallo-enzyme carbonic anhydrase (CA, EC 4.2.1.1)^7^^,^^8^. The ability of sulfocoumarins to selectively inhibit membrane-bound *h*CA IX and XII isoforms were attributed to the unique mechanism of action of these compounds whereby they act as prodrugs activated by CA-mediated hydrolysis^1–6^. This makes these inhibitors fundamentally different from the classical carbonic anhydrase inhibitors (CAIs) – e.g. those of sulphonamide type which act by binding to the CA prosthetic zinc ion present in all isoforms, which makes designing isoform-selective sulphonamide CAIs particularly difficult. On the contrary, CA-mediated hydrolysis of sulfocoumarins **1** (as well as their progenitors coumarins) leads to the *in situ* formation of the *Z*-configured stiryl sulphonic acid (*Z*)-**2** which is likely to isomerise to (*E*)-**2**, the active inhibitor form whose binding to CA was confirmed by X-ray crystallography^1^. This inhibitor activation and binding apparently occurs only in the protein environment of the two membrane-bound isoforms (*h*CA IX and XII) which makes these mechanistically distinct inhibitors ideal tools for targeting hypoxia survival mechanism in tumour cells providing which overexpression of precisely these two isoforms is considered responsible for^9^. Indeed, selective targeting of *h*CA IX and XII has been confirmed to lead to retardation of tumour growth and, ultimately, reduction of tumour size^10^.

Another principal mechanism of tumour survival which we have been recently tackling^11^^,^^12^ as a target for anticancer agent design, is that providing tumour cell defence against oxidative stress (reactive oxygen species or ROS). In particular, tumour cells have been shown to overexpress thioredoxin reductase (TrxR, EC 1.8.1.9) which contributes to their resistant phenotype characterised by higher levels of ROS^13^. Thus, targeting TrxR1 (the most widespread cytosolic isoform of human TrxR) has been investigated as an emerging approach to selective killing of cancer cells^14^. This selenocysteine (Sec) enzyme, along with NADPH and thioredoxin (Trx) is part of the Trx system and responsible for maintaining Trx in its reduced bis-sulfhydryl state. Among several classes of inhibitors of varying degree of electrophilicity towards the catalytic Sec residue (recently reviewed by Bellelli^15^ and Fang^16^), we found covalent Michael acceptor inhibitors (such as Ugi-type adducts **3** which we dubbed “Ugi Michael Acceptors” or UMAs) to be particularly efficacious^12^. The mechanism of inhibitory action of UMAs towards TrxR1 likely involves the irreversible covalent trapping of the selenide group of the catalytic Sec residue (which exists in the ionised form at physiological pH^17^) by the electrophilic β-benzoylacrylamide moiety present in **3**.

**Figure 1.**
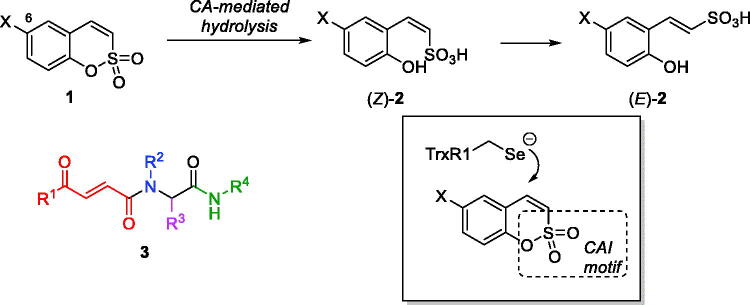
Sulfocoumarins 1 and their CA inhibition mechanism, the previously reported Ugi Michael acceptor TrxR inhibitors (fragments originating from the four components of the Ugi reaction are colour-coded) and the hypothesis for dual CA/TrxR targeting verified in this work.

Considering the presence of a potential Michael acceptor moiety in sulfocoumarins **1**, we hypothesised that in addition to their inhibitory activity towards *h*CAs, these compounds could potentially act as Michael acceptor-type TrxR inhibitors (Figure 1), thus acting as dual inhibitors which target two cancer cell defence mechanisms at a time. Herein, we present our preliminary results obtained in the course of verifying this hypothesis.

## 2. Materials and methods

### 2.1. Chemical syntheses – general

Reagents and starting materials were obtained from commercial sources (Sigma-Aldrich, St. Louis, MO) and used as received. The solvents were purified and dried by standard procedures prior to use; petroleum ether of boiling range 40–60° C was used. Flash chromatography was carried out using Merck silica gel (230–400 mesh). Thin-layer chromatography was performed on silica gel, spots were visualised with UV light (254 and 365 nM). Melting points were determined on an OptiMelt automated melting point system. IR spectra were measured on a Shimadzu FTIR IR Prestige-21 spectrometer. NMR spectra were recorded on Varian Mercury (400 MHz) spectrometer with chemical shifts values (*d*) in ppm relative to TMS using the residual DMSO-d_6_ signal as an internal standard. Elemental analyses were performed on a Carlo Erba CHNSeO EA-1108 apparatus. Starting material sulfocoumarins (**4**^18^ and **5**^19^) were prepared as described previously. Alkynes employed in the synthesis of **1a**–**b** are commercially available. Tetrazoles employed in the synthesis of **1c**–**d** were prepared according to the literature protocols^20^^,^^21^. All reagents for biological assays were purchased from Sigma (St. Louis, MO).

### 2.2. General procedure 1: preparation of sulfocoumarins 1a–b (GP1)

To a solution of **4** (1.0 equiv.) in dry THF (1 mL per mmol of **4**) *N,N*-diisopropylethylamine (DIPEA) (50 equiv.), the appropriate alkyne (1.1, 2.0, or 5.0 equiv.), and CuI (2 equiv.) were added. The resulting mixture was stirred at room temperature under an argon atmosphere for 20 h. Saturated NH_4_Cl was added and extracted with EtOAc, washed with brine and dried over Na_2_SO_4_, and evaporated.

#### 2.2.1. 4-(4-Chlorophenyl)-1-(2,2-dioxido-1,2-benzoxathiin-6-yl)-1H-1,2,3-triazole (1a)

Prepared from **4** (0.15 g, 0.67 mmol), 4-chlorophenylacetylene (0.18 g, 1.34 mmol), CuI (0.26 g, 1.34 mmol), and DIPEA (5.85 mL, 33.6 mmol) according to GP1. Crystallisation from ethanol afforded **1a** as yellow crystalline solid (0.19 g, 77%). Mp 236–237 °C. IR (KBr, cm^−1^) *ν*_max_: 1369 (S–O), 1179 (S–O), and 1169 (S–O). ^1^H NMR (400 MHz, DMSO-d_6_) *δ*: 7.55–7.60 (m, 2H), 7.70 (d, *J*= 10.4 Hz, 1H), 7.75 (d, *J*= 8.9 Hz, 1H), 7.84 (d, *J*= 10.4 Hz, 1H), 7.92–7.97 (m, 2H), 8.12 (dd, *J*= 8.9, 2.7 Hz, 1H), 8.39 (d, *J*= 2.7 Hz, 1H), and 9.38 (s, 1H). ^13^C NMR (100 MHz, DMSO-d_6_) *δ*: 119.9, 120.2, 120.3, 121.4, 123.7, 124.0, 127.0, 128.9, 129.2, 132.9, 134.2, 135.8, 146.4, and 150.1. Anal. Calcd. for C_16_H_10_N_3_O_3_SCl (359.79): C, 53.41; H, 2.80; N, 11.68. Found: C, 53.22; H, 2.79; N, 11.32.

#### 2.2.2. 1-(2,2-Dioxido-1,2-benzoxathiin-6-yl)-4-(4-fluorophenyl)-1H-1,2,3-triazole (1b)

Prepared from **4** (0.15 g, 0.67 mmol), 4-fluorophenylacetylene (0.16 g, 1.34 mmol), CuI (0.26 g, 1.34 mmol), and DIPEA (5.85 mL, 33.6 mmol) according to GP1. Yellow crystalline solid (0.19 g, 80%). Mp 224–225 °C. IR (KBr, cm^−1^) *ν*_max_: 1359 (S–O) and 1179 (S–O). ^1^H NMR (400 MHz, DMSO-d_6_) *δ*: 7.32–7.39 (m, 2H), 7.71 (d, *J*= 10.4 Hz, 1H), 7.75 (d, *J*= 8.9 Hz, 1H), 7.84 (d, *J*= 10.4 Hz, 1H), 7.94–8.00 (m, 2H), 8.12 (dd, *J*= 8.9, 2.6 Hz, 1H), 8.39 (d, *J*= 2.6 Hz, 1H), and 9.33 (s, 1H). ^13^C NMR (100 MHz, DMSO-d_6_) *δ*: 116.1 (d, *J*= 21.9 Hz), 119.8, 119.9, 120.2, 121.4, 123.7, 124.0, 126.6 (d, *J*= 3.2 Hz), 127.4 (d, *J*= 8.3 Hz), 134.2, 135.9, 146.6, 150.1, and 162.4 (d, *J*= 245.3 Hz). Anal. Calcd. for C_16_H_10_N_3_O_3_SF (343.33): C, 55.97; H, 2.94; N, 12.24. Found: C, 55.78; H, 2.94; N, 12.24.

#### 2.2.3. 5-(2,2-Dioxido-1,2-benzoxathiin-6-yl)-1-phenyl-1H-tetrazole (1c)

Compound **5** (0.200 g, 0.649 mmol), 1-phenyl-1,2,3,4-tetrazole^20^ (0.190 g, 1.30 mmol), Cs_2_CO_3_ (0.233 g, 0.714 mmol), CuI (0.124 g, 0.649 mmol), Pd(OAc)_2_ (0.0146 g, 0.0649 mmol), and tris(2-furyl) phosphine (0.030 g, 0.130 mmol) were suspended in dry toluene (3 mL). The mixture was stirred at 40 °C under argon for 20 h, then EtOAc (20 mL) was added and the mixture was filtered through celite. Celite was washed with EtOAc (50 mL). The filtrate and washings were combined and concentrated under reduced pressure. The residue was purified by silica gel chromatography (petroleum ether/EtOAc 2:1) and additionally crystallised from EtOH to give **1c** as yellow crystalline solid (0.076 g, 36%). Mp 189–190 °C. IR (KBr, cm^−1^) ν_max_: 1370 (S–O) and 1178 (S–O). ^1^H NMR (400 MHz, DMSO-d_6_) *δ*: 7.49–7.56 (m, 2H), 7.58–7.68 (m, 6H), 7.78 (d, 1H, *J*= 10.4 Hz), and 8.09–8.12 (m, 1H). ^13^C NMR (100 MHz, DMSO-d_6_) *δ*: 119.2, 119.3, 121.6, 123.6, 126.0, 130.0, 130.8, 131.0, 132.5, 133.8, 135.9, 152.2, and 152.4. Anal. Calcd. for C_15_H_10_N_4_O_3_S (326.33): C,55.21; H, 3.09; N, 17.17. Found: C, 55.25; H, 3.09; N, 17.08.

#### 2.2.4. 1-(2,2-Dioxido-1,2-benzoxathiin-6-yl)-5-(4-fluorophenyl)-1H-1,2,3-triazole (1d)

To a solution of **5** (0.25 g, 1.12 mmol) and 4-fluorophenylacetylene (0.27 g, 2.24 mmol) in dry DMF (0.7 mL), Cp*Ru(PPh_3_)_2_Cl (0.01 mmol) was added and the resulting mixture was stirred at 100 °C under an argon atmosphere for 20 h. The solvent was removed under reduced pressure. The residue was purified by silica gel chromatography (petroleum ether/EtOAc 2:1) to give **1d** as yellow crystalline solid (0.11 g, 28%). Mp 157–158 °C. IR (neat, cm^−1^) *ν*_max_: 1373 (S–O) and 1176 (S–O). ^1^H NMR (400 MHz, DMSO-d_6_) *δ*: 7.25–7.33 (m, 2H), 7.37–7.43 (m, 2H), 7.56 (dd, *J*= 8.8, 2.5 Hz, 1H), 7.61 (d, *J*= 8.8 Hz, 1H), 7.67 (d, *J*= 10.4 Hz, 1H), 7.75 (d, *J*= 10.4 Hz, 1H), 7.97 (d, *J*= 2.5 Hz, 1H), and 8.17 (s, 1H). ^13^C NMR (100 MHz, DMSO-d_6_) *δ*: 116.1 (d, *J*= 22.1 Hz), 119.6, 119.8, 122.4 (d, *J*= 3.2 Hz), 123.7, 127.1, 129.3, 131.1 (d, *J*= 8.8 Hz), 133.4, 133.6, 135.7, 137.0, 150.8, and 162.6 (d, *J*= 247.7 Hz). Anal. Calcd. for C_16_H_10_N_3_O_3_SF (343.33): C, 55.97; H, 2.94; N, 12.24. Found: C, 56.17; H, 2.93; N, 11.93.

### 2.3. Carbonic anhydrase inhibition assay

An Applied Photophysics stopped-flow instrument has been used for assaying the CA catalysed CO_2_ hydration activity^22^. Phenol red (at a concentration of 0.2 mM) has been used as indicator, working at the absorbance maximum of 557 nm, with 20 mM Tris (pH 8.3) as buffer, and 20 mM Na_2_SO_4_ (for maintaining constant the ionic strength), following the initial rates of the CA-catalysed CO_2_ hydration reaction for a period of 10–100 s. The CO_2_ concentrations ranged from 1.7 to 17 mM for the determination of the kinetic parameters and inhibition constants. For each inhibitor, at least six traces of the initial 5–10% of the reaction have been used for determining the initial velocity. The uncatalysed rates were determined in the same manner and subtracted from the total observed rates. Stock solutions of inhibitor (0.1 mM) were prepared in distilled-deionised water and dilutions up to 0.005 nM were done thereafter with the assay buffer. Inhibitor and enzyme solutions were pre-incubated together for 15 min at room temperature prior to assay, in order to allow for the formation of the E–I complex. The inhibition constants were obtained by non-linear least-squares methods using PRISM 3 and the Cheng-Prusoff equation, as reported earlier, and represent the mean from at least three different determinations. All CA isoforms were recombinant ones obtained in-house^23–26^.

### 2.4. TrxR activity by DTNB reduction assay

Determination of TrxR activity in SHSY5Y cell lysate. TrxR activity in cell lysate was measured in 96-well plates using previously described methods^27^^,^^28^. For TrxR activity measurement, compounds of different concentrations were incubated with 50 µg of cell lysate and 200 µM NADPH in a volume of 100 µL of 50 mM Tris–HCl and 1 mM EDTA, pH 7.5 (TE buffer), for different time points in 96-well plates at room temperature. Then, 100 µL of TE buffer containing DTNB and NADPH was added (final concentration: 2.5 mM and 200 µM, respectively), and the linear increase in absorbance at 412 nm during the initial 2 min was measured with a Tecan Infinite M1000 multifunctional microplate reader. TrxR activity was calculated as a percentage of enzyme activity of that of DMSO vehicle treated sample.

### 2.5. Cytotoxicity assay

Thus, monolayer tumour cell lines HT-1080 (human fibrosarcoma), SHSY5Y (human neuroblastoma), and MCF-7 (breast adenocarcinoma) were cultured in standard medium DMEM (Dulbecco’s modified Eagle’s medium) supplemented with 10% foetal bovine serum. About 2000–4000 cells per well (depending on line nature) were placed in 96-well plates and after 24 h compounds were added to the wells. Untreated cells were used as a control. The plates were incubated for 48 h, 37 °C, and 5% CO_2_. The number of surviving cells was determined using 3-(4,5-dimethylthiazol-2-yl)-2,5-diphenyltetrazolinium bromide (MTT). MTT-test: after incubating culture medium was removed and 200 µL fresh medium with 20 µL MTT (2 mg/mL in HBSS) was added in each well of the plate. After incubation (3 h, 37 °C, 5% CO_2_), the medium with MTT was removed and 200 µL DMSO were added at once to each sample. The samples were tested at 540 nm on Thermo Scientific Multiskan EX microplate photometer. The half-maximal inhibitory concentration (IC_50_) of each compound was calculated using Graph Pad Prism^®^ 3.0 (GraphPad Software, La Jolla, CA).

## 3. Results and discussion

### 3.1. Chemistry

**Scheme 1.**
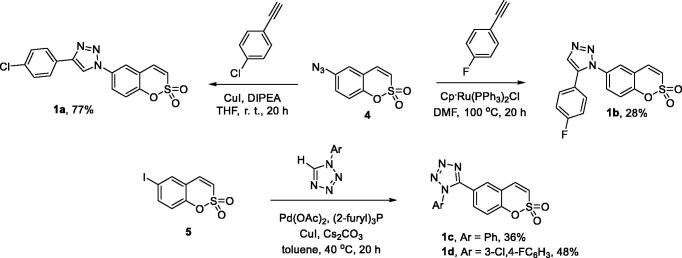
Synthesis of compounds **1a**–**d**.

Compounds **1a** and **1b** were synthesised from azide **4** as described previously^18^. CuI-catalysed Huisgen azide-alkyne cycloaddition gave 1,4-disubstituted 1,2,3-triazole **1a** while employing Ru^II^-catalysed protocol gave 1,5-disusbstituted 1,2,3-triazole **1b**. For the synthesis of 1,5-disubstituted tetrazoles **1c**–**d**, the previously described^19^ Pd-catalysed arylation of 1-aryl tetrazoles with aryl iodide **5** was employed (Scheme 1).

### 3.2. Biological evaluation

To our utmost delight, when the previously established^18^^,^^19^ potent and selective inhibitory profile of compounds **1a**–**d** towards cancer-related *h*CA IX and *h*CA XII isoforms was confirmed in reference to known CAI acetazolamide (**AAZ**), we have also found these compounds to display dose-dependent inhibition of TrxR activity in SHSY5Y cell lysate with IC_50_ values confidently residing in the 10^−5^…10^−4^ M range. Adding to the satisfaction over having our initial hypothesis regarding the dual CA/TrxR inhibitory effects of compounds **1**, rather potent antiproliferative activity was established as evaluated against cultures of cancer cells such as HT-1080 (human fibrosarcoma), SHSY5Y (human neuroblastoma), and MCF-7 (breast adenocarcinoma). These findings are summarised in Table 1.

**Table 1.** Inhibitory profile towards four *h*CA isoforms, TrxR activity in SHSY5Y cell lysate and cytotoxicity towards cancer cell lines determined for compounds **1a**–**d** (nd: not determined).

Compound	IC_50_, μM			TrxR IC_50_, μM	*K_i_*, nM			
	HT-1080	SHSY5Y	MCF-7		*h*CA I	*h*CA II	*h*CA IX	*h*CA XII
**1a**	11.0	22.0	1.8	154	>10,000	>10,000	7.8	17.7
**1b**	8.7	29.0	79.0	40	>10,000	>10,000	8.3	7.8
**1c**	12.3	18.4	101	156	>10,000	>10,000	8.5	7.1
**1d**	7.9	18.5	50.0	125	>10,000	>10,000	6.9	5.4
**AAZ**	nd	nd	nd	nd	250	12	25	5.7

## 4. Conclusions

The previously described sulfocoumarins that were shown to potently and selectively inhibit cancer-related *h*CA IX and *h*CAXII isoforms (whose overexpression is a well-established mechanism of tumour cell defence against hypoxia) also display noticeable, dose-dependent inhibition of TrxR activity in cancer cell lysates. As overexpression of TrxR in cancer cells is a defence mechanism against oxidative stress, the established dual inhibition pattern constitutes a significant starting point for the design and discovery of new anticancer agents based on the dual targeting of the two defence mechanisms crucial for cancer cell survival. This communication opens a new line of research in our laboratories aimed at investigating the practical aspects of the new dual inhibitor design and establishing a well-understood link between inhibition of these two enzyme groups and the dual inhibitors’ antitumor activity. The results of this research will be reported in due course.

## References

[1] Tars K, Vullo D, Kazaks A, et al. Sulfocoumarins (1, 2-benzoxathiine-2, 2-dioxides): a class of potent and isoform-selective inhibitors of tumor-associated carbonic anhydrases. J Med Chem 2013;56:293–300. 23241068 10.1021/jm301625s

[2] Maresca A, Temperini C, Vu H, et al. Non-zinc mediated inhibition of carbonic anhydrases: coumarins are a new class of suicide inhibitors. J Am Chem Soc 2009;131:3057–62. 19206230 10.1021/ja809683v

[3] Touisni N, Maresca A, McDonald PC, et al. Glycosyl coumarin carbonic anhydrase IX and XII inhibitors strongly attenuate the growth of primary breast tumors. J Med Chem 2011;54:8271–7. 22077347 10.1021/jm200983e

[4] Carta F, Maresca A, Scozzafava A, Supuran CT. 5- and 6-Membered (thio)lactones are prodrug type carbonic anhydrase inhibitors. Bioorg Med Chem Lett 2012;22:267–70. 22137345 10.1016/j.bmcl.2011.11.018

[5] Maresca A, Temperini C, Pochet L, et al. Deciphering the mechanism of carbonic anhydrase inhibition with coumarins and thiocoumarins. J Med Chem 2010;53:335–44. 19911821 10.1021/jm901287j

[6] Davis RA, Vullo D, Maresca A, et al. Natural product coumarins that inhibit human carbonic anhydrases. Bioorg Med Chem 2013;21:539–43. 10.1016/j.bmc.2012.07.021 22892213

[7] Alterio V, Di Fiore A, D’Ambrosio K, et al. Multiple binding modes of inhibitors to carbonic anhydrases: how to design specific drugs targeting 15 different isoforms? Chem Rev 2012;112:4421–68. 22607219 10.1021/cr200176r

[8] Supuran CT. Carbonic anhydrases: novel therapeutic applications for inhibitors and activators. Nat Rev Drug Discov 2008;7:168–81. 18167490 10.1038/nrd2467

[9] Schwartz L, Supuran CT, Alfarouk KO. The Warburg effect and the hallmarks of cancer. Anti-Cancer Agents Med Chem 2017;17:164–70. 10.2174/1871520616666161031143301 27804847

[10] Supuran CT. Carbonic anhydrase inhibition and the management of hypoxic tumors. Metabolites 2017;7:48. 10.3390/metabo7030048 PMC5618333 28926956

[11] Bakulina O, Bannykh A, Jovanović M, et al. Design, synthesis, and biological evaluation of novel derivatives of dithiodiglycolic acid prepared via oxidative coupling of thiols. J Enzyme Inhib Med Chem 2019;34:665–71. 30746961 10.1080/14756366.2019.1575372 PMC6374954

[12] Jovanović M, Zhukovsky D, Podolski-Renić A, et al. Novel electrophilic amides amenable by the Ugi reaction perturb thioredoxin system via thioredoxin reductase 1 (TrxR1) inhibition: identification of DVD-445 as a new lead compound for anticancer therapy. Eur J Med Chem 2019;181:111580. 31400708 10.1016/j.ejmech.2019.111580

[13] Zhang J, Li X, Han X, et al. Targeting the thioredoxin system for cancer therapy. Trends Pharmacol Sci 2017;38:794–808. 28648527 10.1016/j.tips.2017.06.001

[14] Arner ES, Holmgren A. The thioredoxin system in cancer. Semin Cancer Biol 2006;16:420–6. 17092741 10.1016/j.semcancer.2006.10.009

[15] Saccoccia F, Angelucci F, Boumis G, et al. Thioredoxin reductase and its inhibitors. Curr Protein Pept Sci 2014;15:621–46. 24875642 10.2174/1389203715666140530091910 PMC4275836

[16] Zhang B, Zhang J, Peng S, et al. Thioredoxin reductase inhibitors: a patent review. Exp Opin Ther Pat 2017;27:547–56. 10.1080/13543776.2017.1272576 27977313

[17] Copeland PR. Making sense of nonsense: the evolution of selenocysteine usage in proteins. Genome Biol 2005;6:221. 15960811 10.1186/gb-2005-6-6-221 PMC1175963

[18] Grandane A, Tanc M, Zalubovskis R, Supuran CT. 6-Triazolyl-substituted sulfocoumarins are potent, selective inhibitors of the tumor-associated carbonic anhydrases IX and XII. Bioorg Med Chem Lett 2014;24:1256–60. 24518190 10.1016/j.bmcl.2014.01.076

[19] Grandane A, Tanc M, Zalubovskis R, Supuran CT. Synthesis of 6-tetrazolyl-substituted sulfocoumarins acting as highly potent and selective inhibitors of the tumor-associated carbonic anhydrase isoforms IX and XII. Bioorg Med Chem Lett 2014;22:1522–8. 10.1016/j.bmc.2014.01.043 24513186

[20] Potewar TM, Siddiqui SA, Lahoti RJ, Srinivasan KV. Efficient and rapid synthesis of 1-substituted-1H-1,2,3,4-tetrazoles in the acidic ionic liquid 1-n-butylimidazolium tetrafluoroborate. Tetrahedron Lett 2007;48:1721–4.

[21] Voitekhovich SV, Vorob'ev AN, Gaponik PN, Ivashkevich OA. Synthesis of new functionally substituted 1-R-tetrazoles and their 5-amino derivatives. Chem Heterocycl Compd 2005;41:999–1004.

[22] Khalifah RG. The carbon dioxide hydration activity of carbonic anhydrase. I. Stop-flow kinetic studies on the native human isoenzymes B and C. J Biol Chem 1971;246:2561–73. 4994926

[23] Maresca A, Carta F, Vullo D, Supuran CT. Dithiocarbamates strongly inhibit the β-class carbonic anhydrases from *Mycobacterium tuberculosis*. J Enzyme Inhib Med Chem 2013;28:407–11. 22145736 10.3109/14756366.2011.641015

[24] Ekinci D, Kurbanoglu NI, Salamci E, et al. Carbonic anhydrase inhibitors: inhibition of human and bovine isoenzymes by benzenesulphonamides, cyclitols and phenolic compounds. J Enzyme Inhib Med Chem 2012;27:845–8. 21999604 10.3109/14756366.2011.621122

[25] Ekinci D, Karagoz L, Ekinci D, et al. Carbonic anhydrase inhibitors: *in vitro* inhibition of α isoforms (*h*CA I, *h*CA II, *h*CA III, *h*CA IV) by flavonoids. J Enzyme Inhib Med Chem 2013;28:283–8. 22168126 10.3109/14756366.2011.643303

[26] Alp C, Maresca A, Alp NA, et al. Secondary/tertiary benzenesulfonamides with inhibitory action against the cytosolic human carbonic anhydrase isoforms I and II. J Enzyme Inhib Med Chem 2013;28:294–8. 22380772 10.3109/14756366.2012.658788

[27] Luthman M, Holmgren A. Rat liver thioredoxin and thioredoxin reductase: purification and characterization. Biochemistry 1982;21:6628–33. 7159551 10.1021/bi00269a003

[28] Arner ES, Zhong L, Holmgren A. Preparation and assay of mammalian thioredoxin and thioredoxin reductase. Meth Enzymol 1999;300:226–39. 10.1016/s0076-6879(99)00129-9 9919525

